# Screening of α-Tocopherol Transfer Protein Sensitive Genes in Human Hepatoma Cells (HepG2)

**DOI:** 10.3390/ijms17071016

**Published:** 2016-06-27

**Authors:** Yang-Hua Qu, Jun-Cai Fu, Kun Liu, Zhao-Yun Zuo, Hui-Na Jia, Yong Ma, Hai-Ling Luo

**Affiliations:** State Key Laboratory of Animal Nutrition, College of Animal Science and Technology, China Agricultural University, Beijing 100193, China; quyanghua@cau.edu.cn (Y.-H.Q.); juncaifu@cau.edu.cn (J.-C.F.); liukun_139@163.com (K.L.); tianai305@163.com (Z.-Y.Z.); jiahuina19880505@126.com (H.-N.J.); syau2010@126.com (Y.M.)

**Keywords:** α-tocopherol transfer protein, vitamin E, sensitive genes, microarray

## Abstract

α-Tocopherol transfer protein (α-TTP) is a ~32 kDa protein expressed mainly in hepatocytes. The major function of the protein is to bind specifically to α-tocopherol and, together, the complex transfers from late lysosomes to the cell membrane. A previous study indicated that some factors might be required in the transferring process. However, there is little information available about the potential transferring factors. In addition, there remains much to learn about other physiological processes which α-TTP might participate in. Thus, in this study a human α-TTP eukaryotic expression vector was successfully constructed and expressed in human hepatoma cells (HepG2). The sensitive genes related to α-TTP were then screened by microarray technology. Results showed that expression of the vector in HepG2 cells led to the identification of 323 genes showing differential expression. The differentially expressed transcripts were divided into four main categories, including (1) cell inflammation; (2) cell cycle and cell apoptosis; (3) cell signaling and gene regulation; and (4) cellular movement. A few cellular movement related transcripts were selected and verified by quantitative real-time PCR. Expressions of some were significantly increased in α-TTP-expressed group, which indicated that these factors were likely to play a role in the transferring process.

## 1. Introduction

Vitamin E was first discovered as a factor crucial for the reproduction of rats; later it was generally considered as an essential lipid-soluble antioxidant [1,2]. Eight different forms of vitamin E exist in nature: α-, β-, γ-, δ-tocopherols with a saturated phytyl side chain, and α-, β-, γ-, δ-tocotrienols with an unsaturated isoprenoid side chain. It is generally accepted that α-tocopherol has the highest biological activity in every variety of form [3]. As a potent antioxidant, vitamin E can prevent lipid peroxidation by scavenging reactive oxygen and nitrogen species [4]. In addition to its antioxidant function, further research suggested that vitamin E played a pivotal role in modulating enzyme activity, cell multiplication, inflammatory response, signal transduction and gene expression [5,6].

Recently, numerous studies have focused on the intracellular transport of vitamin E. In the small intestine, dietary vitamin E is absorbed from epithelial cells and delivered to the liver with chylomicrons [7]. In hepatocytes, the α-tocopherol transfer protein (α-TTP) is responsible for the transport of vitamin E. α-TTP was first described in rat liver cytoplasm in 1975. It is known for its selective binding to α-tocopherol over other forms of vitamin E [8]. The protein binds α-tocopherol with high affinity and catalyzes its transfer from late lysosomes to the cell membrane [9]. It was first purified from rat liver cytoplasm, and then from human liver. Subsequently, the α-TTP gene sequences from both species have also been cloned [10,11,12]. It has received a great deal of attention due to the fact that a mutation of the α-TTP gene was responsible for ataxia with vitamin E deficiency (AVED), resulting, not only in serum vitamin E deficiency, but neurodegeneration as well [13]. Mice carrying a mutated α-TTP gene exhibited symptoms similar to humans with AVED, which even led to infertility [14]. These findings indicated that α-TTP played a key role in transferring α-tocopherol and maintaining its concentration in the plasma at an elevated level.

Extensive research was conducted in rats and humans to clarify whether the level of vitamin E could regulate the expression of α-TTP for decades, but the results seem to be inconclusive. Kim [15] reported that vitamin E supplementation could lower both mRNA and protein expression in rat liver compared with control group, and diets with insufficient vitamin E increased α-TTP mRNA levels, without affecting α-TTP protein concentrations. On the other hand, Shaw [16] suggested that the lack of vitamin E lowered the expression of the α-TTP protein, but had no effects on α-TTP transcripts. Fechner [17] resupplied vitamin E to rats that were previously fed with diets containing insufficient levels of vitamin E for five weeks; this led to an increase in α-TTP mRNA expression. Additional studies later in rat and pig liver, resulted in similar conclusions, namely that vitamin E levels were unrelated to α-TTP expression [18,19,20]. Apart from the experiments in liver tissue, it was also reported in human hepatoma cells by Thakur that treatment with vitamin E would bring about a time- and dose-dependent increase in α-TTP levels [21]. However, it is still difficult to get consistent information from former outcomes.

A series of experiments were conducted in our laboratory in sheep to find out the connection between α-TTP and vitamin E from 2008. We cloned the full-length cDNA sequence of the ovine α-TTP gene, which was added to the Genbank database (ID: NM_001198882) in 2009, and used it to perform bioinformatic analysis. In addition, we generated a monoclonal antibody against ovine α-TTP in 2014. Further studies suggested that high levels of vitamin E supplementation significantly increased ovine α-TTP protein concentrations in the liver. In tissues other than the liver, the expression of the α-TTP gene also increased by the vitamin E supplementation [22,23,24,25]. Nevertheless, there remains much to learn about the α-tocopherol and its transporter. A previous report indicated that some factors contributed to the transport of vitamin E by α-TTP [26], but the information about it was grossly absent. Furthermore, whether there are other physiological processes that α-TTP is involved in or not is poorly studied. Therefore, relevant investigation should be designed to clarify it.

The human hepatoma cell (HepG2) is a commonly used model in vitamin E and α-TTP research, as it is unable to express α-TTP itself in vitro. Expression of TTP in freshly prepared primary hepatocytes declines precipitously following isolation. However, one can transfect TTP gene into the cells by constructing expression vectors to study properties of the protein [9]. In order to illustrate the physiological processes, which α-TTP participates in, and identify transport-related factors, a human α-TTP eukaryotic expression vector was constructed and expressed in HepG2 cells in this experiment. Its sensitive genes were then identified by using microarray technology and their functions were analyzed in a comprehensive way. The results indicated that α-TTP was involved in many different physiological processes. Moreover, a few possible transport-related genes were selected and verified by quantitative real-time PCR. It is speculated that some of them might be required in the transport process.

## 2. Results

### 2.1. Expression of Human α-Tocopherol Transfer Protein (α-TTP) Eukaryotic Expression Vector in Human Hepatoma Cells (HepG2)

**Figure 1:**
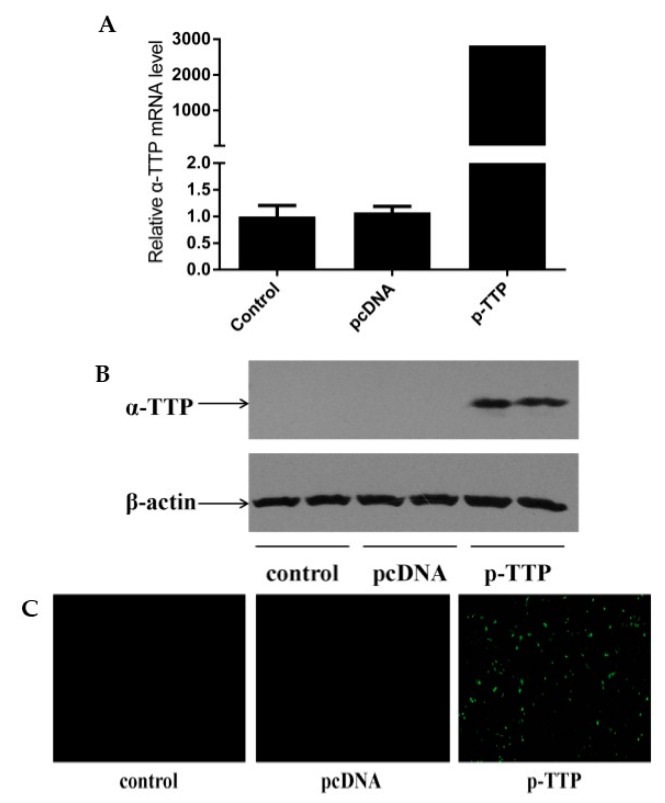
Expression of human α-tocopherol transfer protein (α-TTP) eukaryotic expression vector in Human Hepatoma cells (HepG2). (**A**) Result of quantitative real-time PCR; (**B**) Result of Western blot; (**C**) Result of Immunofluorescence (10×). pcDNA: pcDNA3.1-mycHisa empty vector; p-TTP: human α-TTP eukaryotic expression vector.

Quantitative real-time PCR, Western blot and immunocytochemical analysis all showed that the human α-TTP eukaryotic expression vector was expressed in HepG2 cells with high efficiency, compared with blank control group and empty vector transfection control group (Figure 1). This indicated the successful construction of the expression vector.

### 2.2. Screening Genes Related to α-TTP

The microarray analysis detected 323 differentially expressed genes in cells expressing the human α-TTP eukaryotic expression vector. Of these genes, 256 were upregulated and 67 were downregulated, as compared to the empty vector group. The selected genes were classified after Gene Ontology annotation and Pathway analysis; classification of the α-TTP related genes is shown in Table 1.

### 2.3. Verification of the Transport-Related Genes

**Figure 2:**
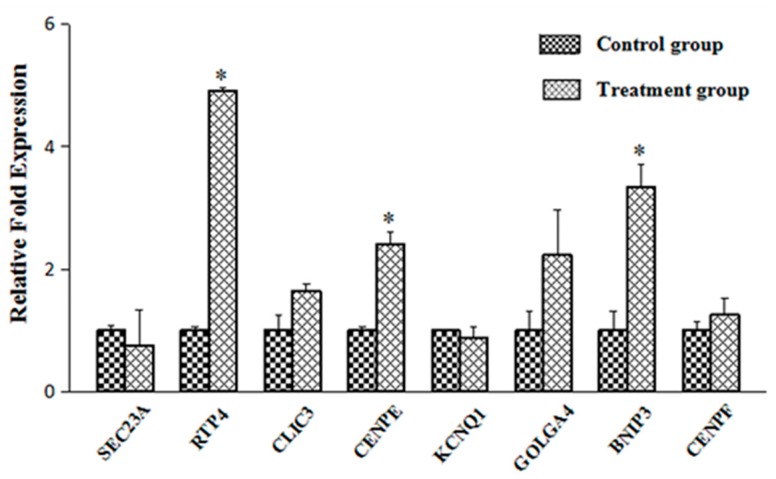
Expression levels of α-TTP related genes. *SEC23A*: Sec23 homolog A; *RTP4*: receptor transporter protein 4; *CLIC3*: chloride intracellular channel 3; *CENPE*: centromere protein E; *KCNQ1*: potassium voltage-gated channel subfamily Q member 1; *GOLGA4*: golgi autoantigen, golgin subfamily a, 4; *BNIP3*: BCL2/adenovirus E1B 19 kDa interacting protein 3; *CENPF*: centromere protein F. Data are presented as mean ± standard error; the asterisk indicated significant difference between control and treatment group.

According to previous studies, α-tocopherol could combine with α-TTP in lysosomes after they got into hepatocytes, then was carried to the cell membranes by α-TTP [9]. However, the detailed process of the transfer remained unclear. One report suggested that α-TTP may need some factors to finish the process [26]. In the present study we hypothesized that certain molecules involved in intracellular movement were likely to regulate the transfer of complexes. A few transcripts were selected after microarray analysis, including Sec23 homolog A (SEC23 A), receptor transporter protein 4 (RTP4), chloride intracellular channel 3 (CLIC3), centromere protein E (CENPE), Potassium voltage-gated channel, KQT-like subfamily (KCNQ1), golgi autoantigen, golgin subfamily a, 4 (GOLGA4), Bcl2/adenovirus E1B 19 kDa interacting protein 3 (BNIP3) and Centormere protein F, 350/400 ka (CENPF), then qRT-PCR was applied to quantitate the expression of these genes. Most gene expression levels in qRT-PCR showed similar trend with microarray data. The result showed that expression levels of these genes related to cellular movement changed to different degrees (Figure 2). The expression of RTP4, CENPE and BNIP3 increased significantly than in the control group. The expression of golgi autoantigen, golgin subfamily a, 4 (GOLGA4) also showed more than two times increase than in the control group.

## 3. Discussion

Recently, α-TTP received extensive attention due to its irreplaceable function in transferring α-tocopherol in hepatocytes. In order to obtain more information about its physiological function and interaction mechanisms with vitamin E, it is necessary to investigate genes that are sensitive to changes in its intracellular concentration. HepG2 cell is a well-known cell line that has no ability to express α-TTP in vitro [9]. In this study, we transfected TTP gene into HepG2 cells by constructing human α-TTP eukaryotic expression vector. Quantitative real-time PCR, Western blot and immunocytochemical analysis were all used to detect the expression. Compared with blank control group and empty vector transfection group, which did not express α-TTP on account of the character of HepG2 cells, the efficient expression in human α-TTP eukaryotic expression vector group indicated the successful construction of this vector, which established the foundation for further research. RNA from HepG2 cells in control group and treatment group was then extracted combined with subsequent microarray analysis. A total of 323 differentially expressed genes (more than 1.5-fold) were identified, including 256 up-regulated and 67 down-regulated ones. After GO annotation and Pathway analysis, these differentially expressed transcripts were divided into four main categories, such as (1) cell inflammation; (2) cell cycle and apoptosis; (3) cell signaling and gene regulation; (4) cellular movement.

It was noted that the inflammatory-response system contained a wide spectrum of mediators. According to one report, α-TTP null mice showed enhanced inflammatory responses, probably as a result of plasma and tissue α-tocopherol deficiency [27]. Another study, using gene expression profiling revealed that a cluster of genes related to inflammation was regulated in the heart tissue of α-TTP null mice. Such genes included immunoglobulin kappa chain and heavy chain, tumor necrosis factor receptor superfamily, chemikine (C–C motif) receptor, chemokine (C–X–C motif) ligand and interleukin-1 receptor-associated kinase 1, suggesting increased inflammatory response in α-TTP^−/−^cardiac tissue on account of lacking α-tocopherol [28]. Present results are compatible with these studies. Three members of the tumor necrosis factor receptor superfamily, well known as a critical factor in eliciting rapid inflammatory events [29], were induced by α-TTP. Chemokine (C–X–C motif) receptor 4, which was upregulated by 1.67-fold, could reduce bronchial inflammation [30]. Interleukin 1 receptor was another gene related to α-TTP that showed a 1.51-fold upregulation. In addition, some related genes, including signal transducer and activator of transcription I, *N*-myc interactor, fibronectin and Leukemia inhibitory factor receptor were also upregulated by 1.65-, 1.70-, 1.62- and 1.58-fold, respectively. These factors mostly participate in different cell inflammatory-response mechanisms [31,32], indicating that α-TTP might also be involved in these processes. Considering the differences in the experimental materials, the results might be better explained by assuming a direct effect of α-TTP on the inflammation-related genes, instead of simply lowering α-tocopherol concentration.

In the present study, we found an indication for altered transcripts related to cell cycle and apoptosis. For example, we detected a 1.56-fold decrease in the mRNA for the regulator of chromosome condensation 1 and a 1.60-fold increase in the mRNA for the checkpoint kinase 2. The former protein is responsible for modulating the formation of the spindle and karyotheca during mitosis, while the latter is known to inhibit the cell cycle [33,34]. From the expression of two transcripts it would be more logic to predict that α-TTP might be involved in blocking the cell cycle process. This prediction is supported by the fact that we also detected a 1.54-fold decrease in the nucleolar and coiled-body phosphoprotein 1 and a 1.73-fold decrease in the eukaryotic translation initiation factor 5A mRNA; these genes play important roles in the regulation of transcription and translation, respectively [35,36]. At the same time, we also detected increased expression of a number of pro-apoptotic genes in cells expressing the human α-TTP eukaryotic expression vector. For example BCL-2 modifying factor, a pro-apoptotic protein found important in cell death signaling pathway [37], was upregulated 1.55-fold. Caspase 7, an executioner protein in cell apoptosis [38] also showed a 1.52-fold change in expression. This finding is in accordance with that of a previous study, in which α-TTP-null mice liver was used; the authors of that report found that numerous genes related to the promotion of cell proliferation and the inhibition of apoptosis were markedly upregulated [39]. These effects, i.e., cell cycle inhibition and cell apoptosis promotion suggested that α-TTP might have anti-cancer function. Vitamin E has previously been shown to be effective in the treatment of certain cancers [40,41,42]. However, it is also possible that α-TTP acts synergistically together with its ligand in the regulation of cell cycle and apoptosis.

According to the present results, the expression of some genes related to cell signaling and gene regulation also changed significantly compared to the control group. For example, Hypoxia-inducible factor 1 (HIF-1) was sensitive to stress signals caused by hypoxia [43]. It was previously reported that transcription of the α-TTP gene in human hepatocytes increased in response to hypoxia treatment [44]. On the contrary, exposure to hyperoxia for 48 h led to a decrease in the expression of α-TTP mRNA in rat liver, while the level of TBARS increased significantly [45]. Our results also showed that HIF-1 was upregulated by 1.71-fold in the human α-TTP eukaryotic expression vector group, which implied that α-TTP could react to oxidative stress. It was suggested that the increased α-TTP expression in response to hypoxia-induced oxidative stress might have triggered the formation of additional carriers to transport vitamin E, and consequently reduced further damage [44]. In addition, Interferon regulatory factor 7, an important signaling molecule that induces interferon formation, which is able to activate the apoptosis pathway and remove inflammatory cells [46], was also upregulated 1.84-fold. We also found a few downregulated transcripts such as Protein arginine methyltransferase and CD3e molecule, epsilon associated protein, which has never been reported before. Further studies are needed to understand these findings.

**Figure 3:**
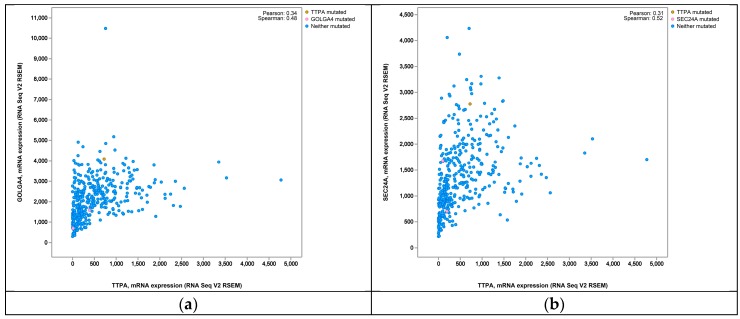
mRNA co-expression in Liver Hepatocellular Carcinoma: (**a**) Tocopherol Transfer Protein A (TTPA) vs. Golgin subfamily a 4 (GOLGA4); (**b**) TTPA vs. Sec24 homolog A (SEC24 A).

Qian [9] demonstrated that α-tocopherol was located in lysosomes together with α-TTP after entering hepatocytes. However, the mechanism by which α-TTP is transferred to the cell surface together with α-tocopherol remains unknown. It is probably that additional factors are also required in this process [26]. Therefore, we have been devoted to searching for some potential elements which contribute to the transfer process. Kono [7] reviewed that phosphoinositide was a kind of possible factor critical for intracellular transport of α-tocopherol, as α-TTP could interact preferentially with phosphatidylinositol 4, 5-bisphosphate in transferring process. PI(4)P is produced by PI-4-kinase in the trans-Golgi. One report showed that PI(4)P would back-transfer from the trans-Golgi to the endoplasmic reticulum (ER) during oxysterol-binding protein (OSBP) transport, which was another family of lipid transfer protein, just like α-TTP [47]. Moreover, some lipoproteins which would be secreted outside the membrane to transfer α-tocopherol in serum were processed through ER-Golgi pathway simultaneously with α-tocopherol transport by α-TTP. We hence speculated that ER-Golgi pathway became more active in the course of transport. In the present study, several genes involved in cellular movement were significantly affected in response to α-TTP expression, including SEC23A (1.51-fold increase), RTP4 (3.13-fold increase), CLIC3 (1.57-fold increase), CENPE (1.72-fold increase), KCNQ1 (1.53-fold increase), GOLGA4 (1.70-fold increase), BNIP3 (1.55-fold increase) and CENPF (1.54-fold increase). SEC23A was once reported as an essential component of vesicles that transport secretory proteins from the ER to the Golgi. A SEC23A mutation would lead to abnormal ER to Golgi trafficking [48]. GOLGA4, also referred to as Trans-Golgi p230, which located in the Golgi apparatus, might play a role in delivery of transport vesicles from the trans-Golgi to plasma membrane [49]. Based on the information in cBioPortal for Cancer Genomics database, the Spearman’s Correlations are 0.48 and 0.52, respectively, in mRNA co-expression: Tocopherol Transfer Protein A (TTPA) vs. GOLGA4 and TTPA vs. SEC24A in Liver Hepatocellular Carcinoma (Figure 3) [50]. It is speculated that these two factors were regulated when α-tocopherol transported by α-TTP, then activated ER-Golgi pathway, finally promoted the transferring process. Besides, other factors were also involved in different transport processes, and some of them were found to be the components of endoplasmic reticulum [51,52,53]. Further validation to these genes by quantitative real-time PCR was conducted, results showed that expression of RTP4, CENPE and BNIP3 were significantly higher than in the control group, and GOLGA4 expression also showed more than two times increase than control; this indicates these genes may play pivotal roles in the transferring process. Research investigating this possibility will soon be conducted.

## 4. Materials and Methods

### 4.1. Construction of a Human Eukaryotic Expression Vector 

First, human α-TTP (GenBank: NM_000370) CDS area gene sequences were synthesized artificially, and Kpn I and Xba I enzyme cutting sites were added in the 5’and 3’ end, respectively. This was used for cloning to the eukaryotic expression vector. The gene sequences were synthesized by Takara Biotechnology (Dalian, China) Co., Ltd.

The human α-TTP eukaryotic expression vector was then constructed. Enzyme digestion reaction was applied to the pcDNA 3.1-mycHisa with Kpn I and Xba I endonucleases. The components were gently mixed and centrifuged, this was followed by incubation in a 37 °C water bath for 1 h. The composition of the enzyme digestion reaction is given in Table 2.

Carrier segments were extracted by agarose gel electrophoresis after enzyme digestion; the extraction reaction was conducted by means of a gel extraction kit (Omega Bio-tek; Norcross, GA, USA). T4 ligases were then used to ligate the human α-TTP CDS area gene sequences and the pcDNA 3.1-mycHisa from the extraction. For ligation the reaction mix was incubated overnight at 16 °C and products were then inserted into *Escherichia coli* followed by extraction of the plasmid from positive colonies. The composition of the ligation reaction is shown in Table 3.

Single colonies were picked and transferred into 1 mL liquid Luria-Bertani (LB) medium; shaking incubation was conducted for 12 h at 37 °C. A PCR reaction was applied to screen positive colonies using the upstream primer 5’-CGCCAGGGTTTTCCCAGTCACGAC-3’ and downstream primer 5’-GAGCGGATAACAATTTCACACAGG-3’. Subsequently, plasmid DNA was extracted by the alkaline lysis method, and was sequenced with the universal primer T7 in Beijing AuGCT DNA-SYN Biotechnology Co., Ltd. (Beijing, China).

### 4.2. Expression of Human α-TTP Eukaryotic Expression Vector in HepG2 Cells

Plasmid DNA was transfected into HepG2 cells. The HepG2 cell line was obtained from the lab of Dr. and Professor Wei-quan Liu (China Agricultural University, Beijing, China). First, normal HepG2 cells were picked up and cultured in 6-well plates. An inverted microscope was used to inspect cells before transfection. The culture medium was removed when the cells reached 90% confluency. The cells were washed twice with DMEM medium and the medium was then removed completely. In two, 1.5-mL centrifuge tubes the following solutions were prepared: in the first tube 4µg plasmid DNA was mixed with 250 µL Opti-MEN^®^ I Reduced Serum Medium (Invitrogen, Carlsbad, CA, USA), while, in the second tube, 10 µL Lipofectamine^®^2000 was mixed with 250 µL Opti-MEN^®^ I Reduced Serum Medium (Invitrogen). The content of the tubes were mixed, the mixture was left for 30 min and dropped onto the cells and the cells were cultured in an environment of 5% CO_2_, at 37 °C for 4–6 h. The liquid was then discarded and the cells were washed with DMEM medium an additional two times. The cells were then cultured in DMEM medium without double resistant containing 10% fetal bovine serum (FBS) in an environment of 5% CO_2_, at 37 °C. A blank control group and an empty vector transfection control group were designed simultaneously.

The human α-TTP eukaryotic expression vector was examined in HepG2 cells with quantitative real-time PCR, western blotting and immunofluorescence. Total RNA was extracted from all groups after 36 h of culture. The primers used for PCR were designed with Primer 5.0, based on human α-TTP sequences (GenBank accession number: NM_000370) and β-actin gene sequences (GenBank: NM_001101). Details of primers for qRT-PCR are given in Table 4.

Total proteins from all groups were extracted after 48 h of culture, and α-TTP protein expression was determined by western-blotting. The primary antibody was human α-TTP monoclonal (Abnova, Taiwan, Dilution ratio 1:1000) and the secondary antibody was goat anti-mouse (Dilution ratio 1:5000) (from Beijing B & M Biotech Co., Ltd., Beijing, China).

Immunofluorescence was also used to examine expression of α-TTP after 48 h of culture. First, the culture medium was discarded. The cells were washed with cold (4 °C) PBS 3 times and 500 µL fixative (propyl alcohol:methyl alcohol, 3:1) was added to each well of a 6-well plate, at 4 °C. The fixative was discarded after 10 min and the cells were washed again with phosphate buffer saline (PBS) 3 times. The primary antibody (monoclonal antibody of human α-TTP, Abnova, Taiwan, 1:100 dilution) was diluted with PBS and 500 µL of the solution was added to each well. The cells were then incubated for 1 h in the dark and washed 3 times with PBS. This was followed by the addition of 500 µL of the secondary antibody (goat-anti-mouse, FITC conjugated; from Beijing Solarbio Technology Co., Ltd., Beijing, China; 1:100 dilution with PBS) to each well. The cells were again incubated for 1 h under in the dark. Finally, they were washed 3 times with PBS and examined using a fluorescence microscope (Olympus, Tokyo, Japan).

### 4.3. RNA Extraction, Microarray Analysis and Statistics

Total RNA from HepG2 cells containing an empty pcDNA3.1-mycHisa vector (control group) and those with a human α-TTP eukaryotic expression vector (treatment group) was extracted and processed for microarray analysis. Genes whose expression changes exceeded 1.5-fold were used for gene ontology (GO) annotation and Pathway analysis to characterize their biological properties.

## 5. Conclusions

In conclusion, our results demonstrate that α-TTP is involved in various physiological processes. Expression of the protein resulted in many differentially expressed transcripts and certain effects were similar to those caused by vitamin E. Moreover, some novel factors were also identified which might have relevance to the transport process of the α-TTP/vitamin E complex, and qRT-PCR was applied to verify their expression. It is possible that some of them may contribute to the transferring process. Nevertheless, further research is still needed to verify it in detail.

## Figures and Tables

**Table 1:** Classification of α-tocopherol transfer protein (α-TTP)-related genes.

Classification	Genbank Accession Number	Name of Gene	Fold Change
Genes related to cell inflammation	NM_004688	*N-myc (and STAT) interactor*	1.70
	NM_001008540	*Chemokine (C–X–C motif) receptor 4*	1.67
	NM_139266	*Signal transducer and activator of transcription 1*	1.65
	NM_002026	*Fibronectin 1*	1.62
	NM_002310	*Leukemia inhibitory factor receptor α*	1.58
	NM_000877	*Interleukin 1 receptor*	1.51
	NM_001065	*Tumor necrosis factor receptor superfamily, member 1A*	1.58
	NM_001561	*Tumor necrosis factor receptor superfamily, member 9*	−2.00
	NM_001190942	*Tumor necrosis factor receptor superfamily, member 10*	3.14
Genes related to cell cycle and cell apoptosis	NM_001030055	*Rho GTPase activating protein 5*	1.51
	NM_022873	*Interferon, α-inducible protein 6*	2.22
	NM_001206701	*SP100 nuclear antigen*	1.74
	NM_001269	*Regulator of chromosome condensation 1*	−1.56
	NM_181558	*Replication factor C (activator 1) 3*	1.52
	NM_005531	*Interferon, γ-inducible protein 16*	1.51
	NM_001003943	*BCL2 modifying factor*	1.55
	NM_001257387	*Checkpoint* *kinase 2*	1.60
	NM_004741	*Nucleolar and coiled-body phosphoprotein 1*	−1.54
	NM_001267058	*Caspase 7, apoptosis-related cysteine peptidase*	1.52
	NM_001143762	*Eukaryotic translation initiation factor 5A*	−1.73
	NM_004052	*BCL2/adenovirus E1B 19 kDa interacting protein 3*	1.55
	NM_001813	*Centromere protein E*	1.72
	NM_016343	*Centromere protein F, 350/400 ka (mitosin)*	1.54
Genes related to cell signaling and gene regulation	NM_004031	*Interferon regulatory factor 7*	1.84
	NM_001243084	*Hypoxia-inducible factor 1*	1.71
	NM_198318	*Protein arginine methyltransferase*	−1.58
	NM_012099	*CD3e molecule, epsilon associated protein*	−1.72
Genes involved in the cellular movement	NM_006364	*Sec23 homolog A*	1.51
	NM_022147	*Receptor transporter protein 4*	3.13
	NM_004669	*Chloride intracellular channel 3*	1.57
	NM_001813	*Centromere protein E*	1.72
	NM_000218	*Potassium voltage-gated channel subfamily* *Q member 1*	1.53
	NM_001172713	*Golgi autoantigen, golgin subfamily a, 4*	1.70
	NM_004052	*BCL2/adenovirus E1B 19 kDa interacting protein 3*	1.55
	NM_016343	*Centormere protein F, 350/400 ka (mitosin)*	1.54

**Table 2:** Composition of the enzyme digestion reaction.

Ingredients	Volume
DNA	≤1 µg
Kpn I	1 µL
Xba I	1 µL
Buffer	5 µL
ddH_2_O	X µL
Total	50 µL

**Table 3:** Composition of the ligation reaction.

Ingredients	Volume
Vector DNA	5 µL
α-TTP DNA	3 µL
Buffer	1 µL
T4 ligase	1 µL
Total	10 µL

**Table 4:** Details of primers used for quantitative real-time PCR.

Name of Genes	Primer Sequence	Product Size (bp)
*α-TTP*	F: ACAGGAGGTAGAAACTCAGCG	104
	R: TCTTCTTGGCTACGGATGGA	
*β-actin*	F: CTCACCGAGCGCGGCTACAG	126
	R: GGAGCTGGAAGCAGCCGTGG	

## Abbreviations

α-TTP	α-tocopherol transfer protein
qRT-PCR	quantitative real-time polymerase chain reaction
SEC23A	sec23 homolog A
RTP4	receptor transporter protein 4
CLIC3	chloride intracellular channel 3
CENPE	centromere protein E
KCNQ1	potassium voltage-gated channel, KQT-like subfamily
GOLGA4	golgi autoantigen, golgin subfamily a, 4
BNIP3	bcl2/adenovirus E1B 19 kDa interacting protein 3
CENPF	centormere protein F, 350/400 ka (mitosin)
